# Practice of ultrasound-guided central venous catheter technique by the French intensivists: a survey from the BoReal study group

**DOI:** 10.1186/s13613-016-0177-x

**Published:** 2016-08-08

**Authors:** Julien Maizel, Marie-Anaïs Bastide, Jack Richecoeur, Eric Frenoy, Christian Lemaire, Bertrand Sauneuf, Hervé Dupont, Fabienne Tamion, Saad Nseir, Damien Du Cheyron, the BoReal Study group, G. Alvado, C. Andrejak, P. Azera, G.. Barjon, C. Boulle, O. Delastre, V. Delerue, M. Detave, P. Dubosq, J. Elbaaj, M. Fiani, P. Jeanjean, F. Lambiotte, O. Leroy, M. Moubarak, R. Pordes, E. Renaud, J. P. Rigaud, A. Rivière, R. Sahri, P. Saint Leger, D. Thevenin, T. Vanderlinden

**Affiliations:** 1 Medical ICU and INSERM U1088, University Hospital, Amiens, France; 2 Medical-Surgical ICU, Medical Center, Beauvais, France; 3 Medical-Surgical ICU, Medical Center, Le Havre, France; 4 Medical-Surgical ICU, Medical Center, Roubaix, France; 5 Medical-Surgical ICU, Medical Center, Cherbourg, France; 6 Department of Anesthesiology and Critical Care and INSERM U1088, University Hospital, Amiens, France; 7 Medical ICU, University Hospital, Rouen, France; 8 Medical ICU, University Hospital, Lille, France; 9 Medical ICU, University Hospital, Caen, France

**Keywords:** Central venous catheter, Ultrasound, Education

## Abstract

**Background:**

The ultrasound (US)-guided technique has been recommended for central venous catheter (CVC) placement in critical care. However, several surveys have shown that the majority of physicians continue to perform landmark procedures. In our region, we have implemented special courses to promote the use of US with formal training and simulators. Ultrasound machines have also been installed in almost every ICU in our area. We designed a survey to investigate whether the training program established for years and the widespread of ultrasound devices in the ICU of our region will be associated with a high rate of physicians performing US procedures.

**Methods:**

A survey comprising 14 questions was designed to elicit information on training in US techniques, the use of US for CVC placement, reasons for nonuse of US and their opinion concerning the need to teach the landmark technique to residents. This survey was electronically sent to every physician of the BoReal study group (32 ICUs located in the North West of France).

**Results:**

We received 190 responses (response rate 66 %) including 34 % of residents. Only 11 % of respondents reported the absence of training in the US technique, and 3 % reported they did not have access to an ultrasound machine. A total of 68 % declared “always” (18 %) or “almost always” (50 %) using US to guide CVC placement. Our results are better than those of previous surveys. The main reasons why physicians did not use the US technique were that they thought that US guidance was unnecessary (36 %) or because the ultrasound machine was not immediately available (33 %). Ninety-one percentages think that the landmark technique should still be taught to the residents. A higher proportion of residents compared to seniors declared that they always or almost always used the US technique.

**Conclusion:**

Training in ultrasound techniques and the widespread availability of ultrasound machines in ICUs seem to improve the rate of US procedures. However, despite strong scientific evidence a proportion of physicians continue to consider the landmark technique as an alternative to US. Training and education are potentially still the best ways to overcome such barriers or conviction.

## Background

For more than 10 years, the ultrasound (US)-guided technique has been recommended for central venous catheter (CVC) placement in critical care patients [1–5]. The superiority of US over the landmarks technique has been demonstrated in terms of success rate, complications, time and economic burden. However, US-guided procedures are far from being systematic, as several surveys have shown that many physicians continue to perform landmark procedures [6–8]. In a prospective study in France, Belgium and Switzerland, only 54 % of central venous lines were placed under ultrasound guidance [9]. More recently, the multicenter randomized controlled trial 3SITES performed in 9 French ICU (4 university hospitals and 5 community hospitals) reported only 67 % of the jugular, 28 % of the femoral and 16 % of the subclavian procedures performed using the ultrasound [10]. The reasons why operators are reluctant to use ultrasound are mainly the absence of training in US techniques, lack of equipment and the belief that US is not necessary [6, 7, 9–12]. The implementation of training session is a key element to increase the rate of US technique. In our region, the US technique has been taught to residents and physicians working in ICU for 10 years. We have implemented special courses to promote the use of US with formal training and simulators. Those training sessions were organized together by the four university hospitals every year in our region. Ultrasound machines have also been installed in almost every ICU in our area. We therefore consider that the great majority of intensivists working in our region are qualified in US procedures and have an ultrasound machine at their disposal, two of the reasons previously reported as obstacles to use the US technique. Consequently, the rate of physicians performing US procedures should be higher in our region than the percentage formerly reported.

We designed a survey to investigate ICU physician CVC placement clinical practices in our region, the reasons why some physicians prefer to use landmarks or US and their opinion concerning the need to continue to teach the landmark technique to residents. The objectives were to observe whether the training program established for years and the widespread availability of ultrasound devices in the ICU of our region will be associated with a high rate of physicians performing US procedures and to list the remaining obstacles persisting against the progression of US technique.

## Methods

### Survey design

A survey comprising 14 questions was developed. Two physicians that are skilled operators were in charge of elaboration of the questions. The survey was constructed using SurveyMonkey^®^ online software (www.surveymonkey.com) according to guidelines [13]. The 14 questions were presented in a single scrolling page. A group of 5 ICU physicians then tested the questionnaire. A test–retest was performed by the same 5 physicians after an interval of 3 weeks to check reliability, as recommended [13]. Questions were designed to elicit information on physician characteristics, experience in CVC placement, training in US techniques, the use of US for CVC placement, reasons for nonuse of US, reasons for use of US and their opinion concerning the need to teach the landmark technique to residents.

### Data collection

The BoReal group is a group of 32 ICUs located in the North West of France (Nord-Pas-de-Calais-Picardie and Normandie regions). This group is composed of 8 university and 24 community ICUs: 5 medical ICUs, 3 surgical ICUs and 24 medical and surgical ICUs. All physicians and residents belonging to the BoReal group provided their e-mail address. Residents had to be currently assigned to the ICU at the time of the survey to be included in the e-mail listing. The survey was sent individually by e-mail to each physician and resident using SurveyMonkey^®^ online software. A cover letter attached to the e-mail briefly explained the study and gave a link to the online survey. Two reminders were sent to nonresponders.

This study was deemed exempt from review by the local ethics committee.

### Statistics

Results are expressed as mean ± SD. Descriptive statistics were used to summarize the data. Qualitative items were compared using a Chi-square test. A *p* value <0.05 was considered to indicate statistically significant differences.

## Results

### Population

A survey was sent by e-mail to a total of 123 residents and 163 senior physicians, 190 of whom responded (global response rate: 66 %) corresponding to 65 residents (response rate: 53 %) and 125 senior physicians (response rate: 77 %). All surveys were complete. The characteristics of the respondents are presented in Table 1.

**Table 1:** Results of the survey in the overall population (*n* = 190)

Age (years)	
<30	29 % (*n* = 55)
30–39	37 % (*n* = 71)
40–49	15 % (*n* = 29)
50–60	15 % (*n* = 28)
>60	4 % (*n* = 7)
Main practice (MR)	
ICU only	84 % (*n* = 159)
OR and ICU	15 % (*n* = 28)
ER and ICU	2 % (*n* = 3)
Practice setting (MR)	
Community hospital	47 % (*n* = 89)
University hospital	59 % (*n* = 112)
Experience in CVC placement (years)	
<1	15 % (*n* = 28)
1–5	27 % (*n* = 51)
6–10	22 % (*n* = 42)
>10	36 % (*n* = 69)
Number of CVCs placed during the last 12 months	
<10 (<1/month)	13 % (*n* = 25)
10–24 (1–2/month)	34 % (*n* = 65)
25–50 (1 per week)	33 % (*n* = 62)
>50 (>1/week)	20 % (*n* = 38)
Preferred site of CVC placement	
Jugular > femoral > subclavian	55 % (*n* = 105)
Jugular > subclavian > femoral	15 % (*n* = 28)
Femoral > jugular > subclavian	7 % (*n* = 13)
Femoral > subclavian > jugular	2 % (*n* = 4)
Subclavian > femoral > jugular	6 % (*n* = 12)
Subclavian > jugular > femoral	15 % (*n* = 28)
Technique learned during residency	
Landmark	50 % (*n* = 94)
Ultrasound	9 % (*n* = 17)
Both	41 % (*n* = 78)
Use of ultrasound for CVC placement	
Always	18 % (35)
Almost always	50 % (94)
Half of the time	17 % (32)
Almost never	10 % (18)
Never	6 % (11)
Reasons why you do not use ultrasound (MR)	
You think you do not need it	36 % (*n* = 68)
Not immediately available	33 % (*n* = 62)
No equipment	3 % (*n* = 6)
You think the procedure is longer with ultrasound	19 % (*n* = 36)
Lack of training	11 % (*n* = 21)
Other reasons	17 % (*n* = 33)
Which ultrasound technique do you use?	
Skin mark	8 % (*n* = 15)
Ultrasound guided	71 % (*n* = 126)
Both	21 % (*n* = 37)
Which situations would encourage you to use ultrasound? (MR)	
Obesity	54 % (*n* = 102)
Clotting abnormalities	64 % (*n* = 122)
Jugular site	45 % (*n* = 85)
Femoral site	19 % (*n* = 37)
Subclavian site	9 % (*n* = 17)
Anatomic difficulties	52 % (*n* = 99)
Missed landmark procedure	43 % (*n* = 81)
None	3 % (*n* = 6)
How many times have you experienced an emergency situation in which ultrasound was not available sufficiently rapidly enough during the last 12 months?	
0	47 % (*n* = 89)
<2	23 % (*n* = 44)
2–5	24 % (*n* = 46)
6–10	5 % (*n* = 9)
>10	1 % (*n* = 2)
Do you think the landmark procedure should still be taught to residents?	
Yes	91 % (*n* = 173)
No	9 % (*n* = 17)

*CVC* central venous catheter, *ER* emergency room, *ICU* intensive care unit, *MR* multiple responses, *OR* operating room

**Fig. 1:**
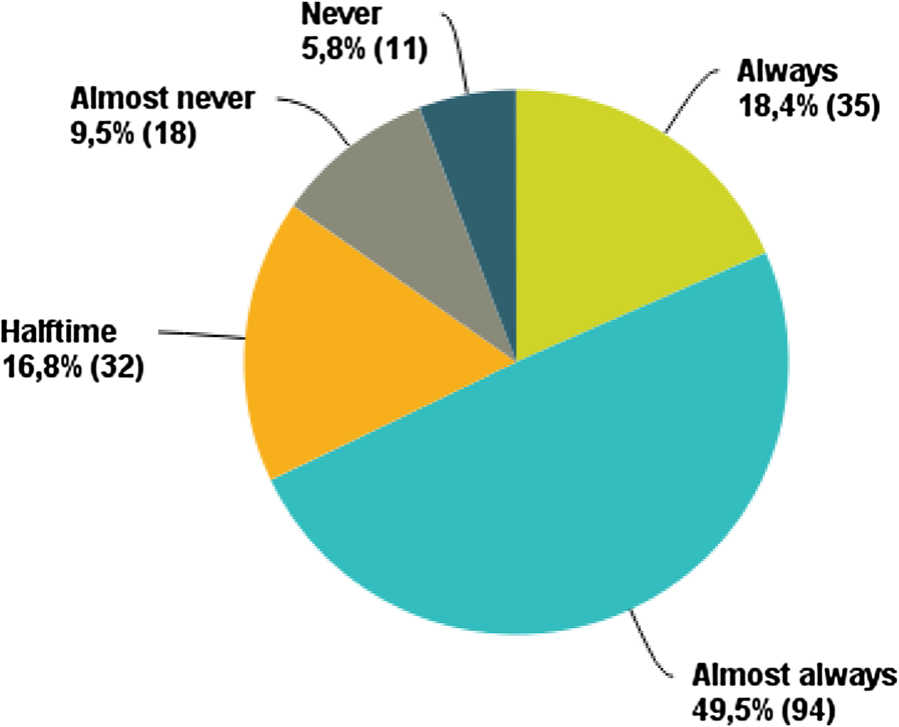
Use of ultrasound for central venous catheter placement in the overall population (*n* = 190)

Only 11 % of respondents reported the absence of training in the US technique, and 3 % reported they did not have access to an ultrasound machine. A total of 68 % declared “always” (18 %) or “almost always” (50 %) using US to guide CVC placement. About 6 % never and 10 % almost never used the US-guided technique (Fig. 1). The main reasons why physicians did not use the US technique were that they thought that US guidance was unnecessary (36 %) or because the ultrasound machine was not immediately available (33 %) (Table 1). Clotting abnormalities (64 %), obesity (54 %) and anatomic difficulties (52 %) were the main situations that led the physician to use ultrasound for CVC placement.

Fifty-three percent of physicians declared that they had experienced an emergency situation in which they could not wait for the ultrasound machine at least once during the last year, and 91 % considered that the landmark technique should still be taught to residents (Table 1).

### Comparisons of residents and seniors

We compared the results of the survey between residents (*n* = 65) and senior physicians (*n* = 125) to determine whether younger physicians behave differently from senior physicians.

The comparison of demographic characteristics between residents and seniors is presented in Table 2. Senior physicians compared to residents preferred more frequently the subclavian site [respectively, 29 % (*n* = 36) vs. 6 % (*n* = 4); *p* = 0.01], while residents tended to prefer the jugular site [61 % (*n* = 76) vs. 88 % (*n* = 57); *p* = 0.01]. The majority of senior physicians only learned the landmark technique (73 %) during their residency training, but this rate dropped to only 5 % for the current residents (*p* = 0.001).

**Table 2:** Comparison of the residents and senior physician answers to the first 7 questions of the survey (demographic data)

	Residents *n* = 65	Seniors *n* = 125	*p* value
Age (years)			
<30	82 % (*n* = 53)	2 % (*n* = 2)	0.001
30–39	18 % (*n* = 12)	47 % (*n* = 59)	0.001
40–49	0	23 % (*n* = 29)	–
50–60	0	22 % (*n* = 28)	–
>60	0	6 % (*n* = 7)	–
Main practice			
ICU only	61 % (*n* = 40)	95 % (*n* = 119)	0.001
OR and ICU	37 % (*n* = 24)	3 % (*n* = 4)	0.001
ER and ICU	2 % (*n* = 1)	2 % (*n* = 2)	0.6
Practice setting (MR)			
Community hospital	78 % (*n* = 51)	49 % (*n* = 61)	0.002
University hospital	34 % (*n* = 22)	54 % (*n* = 67)	0.01
Experience in CVC placement (years)			
<1	42 % (*n* = 27)	1 % (*n* = 1)	0.001
1–5	58 % (*n* = 38)	10 % (*n* = 13)	0.001
6–10	0	34 % (*n* = 42)	–
>10	0	55 % (*n* = 69)	–
Number of CVCs placed during the last 12 months			
<10 (<1/month)	8 % (*n* = 5)	16 % (*n* = 20)	0.2
10–24 (1–2/month)	35 % (*n* = 23)	34 % (*n* = 42)	0.9
25–50 (1 per week)	40 % (*n* = 26)	29 % (*n* = 36)	0.2
>50 (>1/week)	17 % (*n* = 11)	22 % (*n* = 27)	0.6
Preferred site of CVC placement			
Jugular > femoral > subclavian	81 % (*n* = 53)	42 % (*n* = 52)	0.001
Jugular > subclavian > femoral	6 % (*n* = 4)	19 % (*n* = 24)	0.03
Femoral > jugular > subclavian	6 % (*n* = 4)	7 % (*n* = 9)	0.9
Femoral > subclavian > jugular	0	3 % (*n* = 4)	–
Subclavian > femoral > jugular	0	10 % (*n* = 12)	–
Subclavian > jugular > femoral	6 % (*n* = 4)	19 % (*n* = 24)	0.03
Technique learned during residency			
Landmark	5 % (*n* = 3)	73 % (*n* = 91)	0.001
Ultrasound	25 % (*n* = 16)	1 % (*n* = 1)	0.001
Both	71 % (*n* = 46)	26 % (*n* = 32)	0.001

*CVC* central venous catheter, *ER* emergency room, *ICU* intensive care unit, *MR* multiple responses, *OR* operating room

**Fig. 2:**
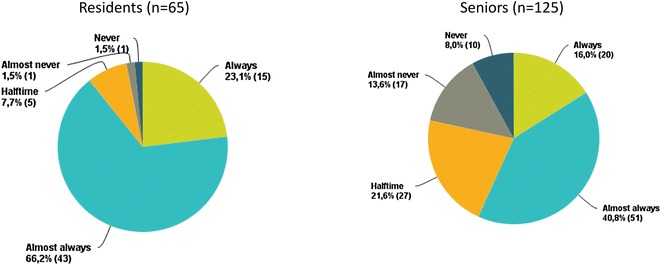
Use of ultrasound for central venous catheter placement among residents (*n* = 65) and senior physicians (*n* = 125)

The proportion of physicians who always used US was not different between the residents and seniors [respectively, 23 % (*n* = 15) vs. 16 % (*n* = 20); *p* = 0.3] (Fig. 2; Table 3). However, a higher proportion of residents declared that they “always” or “almost always” used the US technique compared with the seniors [respectively, 89 % (*n* = 58) vs. 57 % (*n* = 71); *p* = 0.01]. Therefore, although residents and seniors both continue to perform landmark procedures, residents more frequently used US guidance than seniors. Unavailability of the ultrasound machine was reported significantly more frequently as a reason for using the landmark procedure by residents than by senior physicians [43 % (*n* = 28) vs. 27 % (*n* = 34); *p* = 0.04]. Subclavian CVC placement was more frequently reported as the reason for using US by residents compared to seniors [respectively, 17 % (*n* = 11) vs. 5 % (*n* = 6); *p* = 0.01]. The very great majority of the two groups reported that the landmark technique should still be taught to residents.

**Table 3:** Comparison of the residents and senior physician answers to the last 6 questions of the survey (use of the ultrasound technique)

	Residents *n* = 65	Seniors *n* = 125	*p* value
Use of ultrasound for CVC placement			
Always	23 % (*n* = 15)	16 % (*n* = 20)	0.3
Almost always	66 % (*n* = 43)	41 % (*n* = 51)	0.001
Half of the time	8 % (*n* = 5)	22 % (*n* = 27)	0.03
Almost never	2 % (*n* = 1)	14 % (*n* = 17)	0.01
Never	2 % (*n* = 1)	8 % (*n* = 10)	0.1
Reasons you do not use ultrasound (MR)			
You think you do not need it	28 % (*n* = 18)	40 % (*n* = 50)	0.1
Not available	43 % (*n* = 28)	27 % (*n* = 34)	0.04
No equipment	9 % (*n* = 6)	0	–
You think the procedure is longer with ultrasound	14 % (*n* = 9)	22 % (*n* = 27)	0.3
Lack of training	5 % (*n* = 3)	14 % (*n* = 18)	0.07
Other reasons	8 % (*n* = 5)	22 % (*n* = 28)	0.02
Which ultrasound technique do you use?			
Skin mark	3 % (*n* = 2)	11 % (*n* = 13)	0.13
Ultrasound guided	81 % (*n* = 52)	65 % (*n* = 74)	0.007
Both	16 % (*n* = 10)	24 % (*n* = 27)	0.4
Which situations would encourage you to use ultrasound? (MR)			
Obesity	57 % (*n* = 37)	52 % (*n* = 65)	0.6
Clotting abnormalities	65 % (*n* = 42)	64 % (*n* = 80))	0.9
Jugular site	51 % (*n* = 33)	42 % (*n* = 52	0.3
Femoral site	12 % (*n* = 8)	23 % (*n* = 29)	0.1
Subclavian site	17 % (*n* = 11)	5 % (*n* = 6)	0.01
Anatomic difficulties	57 % (*n* = 37)	50 % (*n* = 62)	0.4
Missed landmark procedure	37 % (*n* = 24)	46 % (*n* = 57)	0.3
None	0	5 % (*n* = 6)	–
How many times have you experienced an emergency situation in which ultrasound was not available sufficiently rapidly during the last 12 months?			
0	40 % (*n* = 26)	50 % (*n* = 63)	0.2
<2	35 % (*n* = 23)	17 % (*n* = 21)	0.007
2–5	21 % (*n* = 14)	26 % (*n* = 32)	0.6
6–10	3 % (*n* = 2)	6 % (*n* = 7)	0.7
>10	0	2 % (*n* = 2)	–
Do you think the landmark procedure should still be taught to residents?			
Yes	95 % (*n* = 62)	89 % (*n* = 111)	0.4
No	5 % (*n* = 3)	11 % (*n* = 14)	0.2

*CVC* central venous catheter, *MR* multiple responses

## Discussion

All guidelines recommend the use of US for CVC placement because US has been shown to increase successful catheter placement and to reduce complications [1–5]. General barriers to ultrasound-guided CVC include access to equipment and proper training. In our region we have implemented special courses to promote the use of US with formal training and simulators which should be associated with a high rate of US procedures. Our results are encouraging as 68 % of our physicians use “always” or “almost always” the US. In 2007, Bailey et al. [6] reported only 15 % of cardiovascular anesthesiologists in the USA who performed always or almost always US technique. Moreover, the 18 % of our physicians declaring systematically using US is higher than the 5 % reported by Mimoz et al. [14] in a population of French intensivists in 2006. The proportion of physicians who never used US was lower (6 %) in our study compared to previous studies (44 % in the study by Buchanan et al. [12]). Therefore, our results are better than those of previous surveys. We compared residents and senior physicians to identify current trends in the use of US. This comparison confirmed a trend toward increased use of US among younger physicians who also reported less frequently a lack of training to justify the use of landmark technique (Table 3).

The first reason reported by the physicians in our survey for not using US was “they don’t need ultrasound” (36 %) (Table 1). The same reason of “no apparent need” has already been reported in previous surveys [6, 7]. Despite more than 5 national and international guidelines recommending the use of US, some physicians still consider landmark procedures as a reasonable alternative. More than 40 studies and 7 meta-analyses have shown a clear benefit of using US in terms of success rate and complications. No study has ever demonstrated the superiority of landmark technique over the US-guided technique. However, a proportion of physicians may still consider the benefit of using US to be very minor. It is noteworthy that our survey showed that the subclavian site was the site associated with the lowest rate of US guidance (9 %) compared with the jugular (45 %) and femoral (19 %) sites (Table 1). Although, historically, the jugular vein has been most extensively studied, several studies have now also demonstrated the benefits of US for femoral and subclavian CVC placement, which is why US is now recommended in all sites. For example, in a study based on more than 400 subclavian procedures, Fragou et al. [15] demonstrated the superiority of the US-guided technique over the landmark technique in terms of success rate, number of punctures, complications and procedure time. The recent meta-analysis published by the Cochrane database showed a benefit of US for the jugular site in terms of success rate, complication rate and procedure time [16]. For the subclavian vein, US provides a benefit in terms of arterial puncture and hematoma, but not in terms of success rate and only a benefit in terms of success rate is observed for the femoral site [17]. As stated by the authors of the review, fewer studies have been conducted on the subclavian and femoral veins compared to the jugular vein and the studies included in this meta-analysis were very heterogeneous. More than 5 additional studies on subclavian or femoral vein CVC placement, published since this meta-analysis, have confirmed the superiority of US. Interestingly, our survey shows that residents use more frequently the US for subclavian site than the seniors (Table 3). It is highly possible that in the next years the rate of US subclavian CVC will increase.

In previous surveys, lack of training and absence of an ultrasound machine were cited as the main reasons for not using US [7]. In our region, only 11 % of physicians reported the absence of US training and 3 % reported absence of an ultrasound machine to explain the use of the landmark procedure, which could explain the more frequent use of US procedures in our region compared to previous surveys. However, availability of an ultrasound machine was still a limiting factor for residents, who more frequently reported inaccessibility of the ultrasound machine than seniors to explain use of the landmark technique (Table 3).

Ninety-one percent of physicians recommended teaching the landmark technique, indicating that a large proportion of the physicians who always use US guidance believe that physicians should still be able to perform CVC placement without US. What can justify to perform a landmark procedure if you have an US machine in your setting and you have been trained to US procedures? The only situation remaining is an extreme urgent situation. Fifty-three percent of physicians reported having experienced an emergency situation in which they were unable to wait for the ultrasound machine at least once during the last year. Such situations could justify teaching the landmark technique as a rescue technique, especially as we have demonstrated that residents who have only learned the US-guided technique are unable to perform a landmark procedure [18]. But teaching the landmark procedure during emergency situations where you do not have the time to wait for the US (cardiac arrest for example) is not recommended. The residents will have to perform and learn landmark procedures in nonemergency patients which is associated with a high rate of complications and low success rate during their learning curve. The use of intraosseous catheter could be a good alternative to CVC in case of extreme emergency as previously reported [19, 20].

Our survey presents several limits. The survey responses were self-reported; thus, unknown errors or bias may have resulted from this type of study. In an attempt to keep survey questions brief, questions may have been ambiguous and may have been misinterpreted from their original intent. However, the pilot testing and the test–retest performed by 5 physicians were used to improve the questionnaire as recommended [13]. Our response rate of 66 % may be considered insufficient increasing the risk that our results differ from the nonrespondents. But a response rate between 50 and 60 % is usually considered as acceptable, and the mean response rate reported for physicians in published surveys is between 54 and 61 % [13, 21].

In conclusion, training in ultrasound techniques and the widespread availability of ultrasound machines in ICUs seem to improve the rate of US procedures. However, a proportion of physicians still continue to perform landmark techniques and consider this technique as an alternative to US. The translation of evidence to clinical practice regarding the benefits of ultrasound guidance for central venous catheter placement faces many barriers. The belief that they do not need US despite strong scientific evidence and the desire to continue to teach the landmark technique to residents indicate the aspiration of physicians to continue landmark procedures. Training and education are potentially still the best ways to overcome such barriers or conviction.

## Abbreviations

CVC: central venous catheter
ER: emergency room
ICU: intensive care unit
OR: operating room
US: ultrasound
